# HMB-Containing Oral Nutritional Supplementation and Mortality After Hip Fracture in Malnourished Older Adults: A Formulation-Specific Subanalysis of a Prospective Cohort

**DOI:** 10.3390/nu18121891

**Published:** 2026-06-11

**Authors:** Francisco José Sánchez-Torralvo, Verónica Pérez-del-Río, Luis Ignacio Navas Vela, María García-Olivares, Nuria Porras, Jose Abuín Fernández, Gabriel Olveira

**Affiliations:** 1 Unidad de Gestión Clínica de Endocrinología y Nutrición, Hospital Regional Universitario de Málaga, 29007 Malaga, Spaingabrielm.olveira.sspa@juntadeandalucia.es (G.O.); 2 Instituto de Investigación Biomédica de Málaga (IBIMA), Plataforma Bionand, 29010 Malaga, Spain; 3 Departamento de Medicina y Dermatología, Facultad de Medicina, University of Malaga, 29010 Malaga, Spain; veronicaperezdelrio@gmail.com; 4 Unidad de Gestión Clínica de Cirugía Ortopédica y Traumatología, Hospital de la Axarquía, 29010 Malaga, Spain; 5 Unidad de Gestión Clínica de Endocrinología y Nutrición, Hospital Universitario Torrecárdenas, 04009 Almería, Spain; 6 Centro de Investigación Biomédica en Red de Diabetes y Enfermedades Metabólicas Asociadas (CIBERDEM), Instituto de Salud Carlos III, 28029 Madrid, Spain

**Keywords:** hip fracture, malnutrition, oral nutritional supplements, β-hydroxy-β-methylbutyrate, mortality, older adults, treatment persistence, nutritional support

## Abstract

Background: Oral nutritional supplementation (ONS) is commonly prescribed in malnourished older adults after hip fracture, but formulations are heterogeneous, and their comparative association with mortality remains unclear. We aimed to evaluate whether HMB-containing ONS was associated with lower mortality than non-HMB ONS and to explore whether supplement formulation combined with treatment persistence was associated with differential mortality patterns. Methods: This was a formulation-specific subanalysis of a previously described prospective cohort of older adults with hip fracture and malnutrition or significant nutritional risk. Only patients with known ONS formulation were included (*n* = 107): 59 received HMB-containing ONS, and 48 received non-HMB ONS, including standard, diabetes-specific, and renal-oriented formulations. Mortality at 3, 6, and 12 months was analyzed using crude comparisons and multivariable logistic regression adjusted for sex, age, and Charlson comorbidity index. A 6-month adjusted Cox model was used as the main time-to-event analysis. Exploratory analyses assessed mortality according to supplement formulation and treatment persistence. Results: Overall mortality was 14.0% at 3 months, 23.4% at 6 months, and 29.9% at 12 months. At 6 months, mortality was lower among patients receiving HMB-containing ONS than among those receiving non-HMB ONS (13.6% vs. 35.4%; *p* = 0.011), and the association remained significant after adjustment (OR 0.267; 95% CI 0.091–0.784; *p* = 0.016). Associations at 3 and 12 months were directionally consistent but not statistically significant. In the adjusted Cox model, prescription of HMB-containing ONS was associated with a lower hazard of death within 6 months (HR 0.358; 95% CI 0.145–0.885; *p* = 0.026). Exploratory analyses showed a 6-month mortality gradient according to formulation and persistence, ranging from 0.0% in patients receiving HMB-ONS for ≥3 months to 41.2% in those receiving non-HMB ONS for <3 months. Conclusions: In this formulation-specific subanalysis of malnourished older adults with hip fracture, an association between HMB-containing ONS and lower 6-month mortality was observed compared with non-HMB ONS. Exploratory findings suggested a clinically relevant mortality gradient according to both supplement formulation and treatment persistence, although these results should be interpreted cautiously. Larger prospective studies are warranted to confirm these findings.

## 1. Introduction

Hip fracture in older adults remains a major cause of morbidity, functional decline, institutionalization, and mortality [1,2]. Older patients with hip fractures frequently present with frailty, multimorbidity, and a high prevalence of malnutrition or nutritional risk, which may adversely affect recovery, functional outcomes, and survival [1,2,3,4]. Accordingly, nutritional status has emerged as a clinically relevant and potentially modifiable determinant of outcomes after hip fracture [2,3].

Oral nutritional supplementation (ONS) is commonly prescribed in malnourished older adults after hip fracture as part of perioperative and post-discharge care [1,5,6]. However, despite its widespread use and biological plausibility, available evidence for a consistent survival benefit remains heterogeneous [5,7,8]. Randomized and interventional studies have reported that ONS may improve nutritional intake, nutritional parameters, and selected short-term recovery-related outcomes after hip fracture [9,10,11,12]. In addition, meta-analytic evidence suggests beneficial effects on postoperative complications, including infective complications, pressure ulcers, and overall complications [7,8]. Nevertheless, the effect of ONS on mortality remains uncertain across studies, particularly in the context of heterogeneous patient populations, treatment duration, adherence, and real-world implementation [5,7,13,14]. More recent clinical studies also support a potential benefit of perioperative or postoperative ONS on selected short-term clinical outcomes, although the impact on longer-term hard endpoints remains less well established [12,15].

In a previous analysis of our prospective hip fracture cohort, sustained ONS use for at least 3 months was associated with lower mortality among malnourished patients [16]. This observation supports the clinical relevance of treatment persistence in this setting and is consistent with the notion that the effectiveness of nutritional support may depend not only on whether ONS is prescribed, but also on treatment duration and continuity [7,8,14]. However, ONS should not be considered a homogeneous exposure, as studies in this field have evaluated formulations with differing nutritional composition and intervention characteristics [5,7,8]. In routine clinical practice, the choice of ONS formulation may also be influenced by the patient’s nutritional profile and relevant comorbidities. Therefore, whether outcomes differ according to the specific type of ONS prescribed remains an unresolved and clinically relevant question.

Among the available formulations, HMB-containing ONS may be of particular interest in older adults recovering from hip fracture. β-hydroxy-β-methylbutyrate (HMB), a leucine metabolite, has been associated with anti-catabolic and muscle-preserving effects, which may be especially relevant in the acute post-fracture period, characterized by immobilization, inflammation, and accelerated muscle loss [17,18,19,20,21]. In older adults with hip fracture, studies evaluating HMB-enriched nutritional supplementation have reported improvements in nutritional recovery, muscle-related and functional outcomes, as well as in selected postoperative clinical outcomes [17,18,22]. However, the available evidence remains limited and has focused mainly on intermediate clinical endpoints rather than survival [17,22]. To date, data specifically evaluating whether HMB-enriched ONS is associated with improved survival compared with other ONS formulations in malnourished hip fracture patients remain scarce.

We hypothesize that, among malnourished older adults with hip fractures receiving ONS, exposure to an HMB-containing formulation is associated with lower mortality than exposure to non-HMB ONS. Therefore, the aim of the present study is to evaluate, in a formulation-specific subanalysis of a previously described prospective cohort, whether HMB-containing ONS is associated with lower mortality compared with non-HMB ONS, and to explore whether the combination of supplement formulation and treatment persistence is associated with differential mortality patterns during follow-up.

## 2. Materials and Methods

### 2.1. Study Design and Population

This was a prospective observational subanalysis of a previously described cohort of older adults admitted for fragility hip fracture to the Trauma Surgery Unit of a tertiary care hospital (Regional Hospital of Málaga, Málaga, Spain) between September 2019 and February 2021. The design and main characteristics of the parent cohort have been reported elsewhere. Briefly, the original cohort included patients aged 65 years or older hospitalized for proximal femur fracture due to low-energy trauma, such as a fall from standing height or less. Patients with high-energy fractures, pathologic fractures, periprosthetic fractures, or terminal illness were excluded according to the original study protocol.

For the present subanalysis, we included only patients from the malnourished subgroup of the original cohort who had a recorded prescription of oral nutritional supplementation (ONS) and in whom the specific ONS formulation could be identified from the prescription and dispensing records. Patients with unknown or unavailable ONS formulation were excluded from the formulation-specific analysis.

This study was reported in accordance with the Strengthening the Reporting of Observational Studies in Epidemiology (STROBE) guidelines.

### 2.2. Clinical and Nutritional Assessment

Baseline clinical and functional variables were collected prospectively during the index hospitalization according to the original study protocol. Demographic data, fracture history, and length of hospital stay were recorded. Medical comorbidity burden was assessed using the Charlson Comorbidity Index (CCI). Pre-fracture functional status was evaluated using the Barthel Index and the Functional Ambulation Category (FAC) scale.

Nutritional status was assessed within the first 24–48 h after surgery by the Nutrition Unit as part of routine clinical practice using the Subjective Global Assessment (SGA), which was the prespecified nutritional assessment tool in the parent ONS study. Patients were classified as well nourished, moderately malnourished, or severely malnourished according to SGA. In the present subanalysis, only patients from the malnourished subgroup who were prescribed ONS were considered. When available, body mass index (BMI), serum albumin, and baseline MNA-SF were also recorded and were used for descriptive purposes.

### 2.3. Oral Nutritional Supplementation Classification and Exposure Groups

In the parent cohort, ONS was prescribed according to usual clinical practice to patients diagnosed with severe malnutrition or with moderate malnutrition in the presence of complications or reduced oral intake, based on SGA. The oral nutritional supplements evaluated in this study were commercially available products routinely prescribed in clinical practice and dispensed through routine outpatient pharmacy services according to standard clinical indications. No nutritional products were provided specifically for this study by any commercial company. For the present subanalysis, ONS exposure was classified according to supplement formulation and dispensing persistence.

The main exposure variable for the primary analysis classified patients into two groups according to the prescribed ONS formulation: HMB-containing ONS and non-HMB ONS. In this cohort, HMB-containing ONS corresponded to a high-calorie, high-protein oral nutritional supplement enriched with β-hydroxy-β-methylbutyrate (HMB), whereas non-HMB ONS comprised a heterogeneous group of formulations, including both standard supplements and disease-specific products. The latter consisted predominantly of diabetes-specific and renal-oriented formulations prescribed according to clinical indication, reflecting routine clinical practice.

Treatment persistence was assessed using electronic pharmacy dispensing records. These records captured ONS retrieval from the hospital pharmacy but did not provide information on actual consumption, daily dose, number of servings consumed, adherence to the prescribed regimen, or reasons for treatment discontinuation. Consistent with the parent ONS study, sustained ONS use was defined as dispensing for 3 months or longer, whereas non-sustained use included patients with dispensing for less than 3 months or without effective dispensing after prescription.

For exploratory analyses, patients were additionally categorized into four groups according to supplement formulation and treatment persistence: (1) HMB-ONS for ≥3 months, (2) HMB-ONS for <3 months, (3) non-HMB ONS for <3 months, and (4) non-HMB ONS for ≥3 months.

### 2.4. Follow-Up and Outcomes

Follow-up was performed at 3, 6, and 12 months after the index hip fracture. Mortality data were obtained from the electronic medical records and institutional follow-up procedures used in the parent cohort.

The main clinical outcome of this subanalysis was all-cause mortality after hip fracture. Mortality at 3, 6, and 12 months was analyzed in the binary comparison between HMB-containing ONS and non-HMB ONS. Time-to-event analyses were subsequently focused on the first 6 months after fracture, as this interval showed the clearest separation between groups in the primary comparison and was therefore selected as the focus of the exploratory survival analyses.

### 2.5. Statistical Analysis

Quantitative variables are presented as mean ± standard deviation (SD), and qualitative variables as absolute frequency and percentage. Baseline characteristics were compared between patients receiving HMB-containing ONS and those receiving non-HMB ONS using Student’s *t*-test or the Mann–Whitney U test for continuous variables, as appropriate, and the chi-square test or Fisher’s exact test for categorical variables.

For the primary analysis, the association between ONS formulation (HMB-containing ONS vs. non-HMB ONS) and mortality at 3, 6, and 12 months was evaluated using crude comparisons and multivariable logistic regression models adjusted for sex, age, and Charlson Comorbidity Index. These covariates were selected based on clinical relevance and previous literature. Sensitivity analyses for 6-month mortality were performed using alternative multivariable logistic regression models in which the Charlson comorbidity index was replaced by diabetes mellitus or renal impairment as clinically relevant covariates, given their imbalance between supplement groups and their potential influence on ONS formulation selection.

Time-to-event analysis for 6-month mortality was performed using Cox proportional hazards regression adjusted for sex, age, and Charlson Comorbidity Index. Adjusted survival curves derived from the Cox model were generated for visual representation of the main association. The proportional hazards assumption was assessed graphically using log-minus-log survival plots.

For exploratory analyses, 6-month mortality was also examined across the four groups defined by supplement formulation and treatment persistence. Crude overall comparisons across the four groups were performed using the chi-square test, and selected pairwise comparisons were assessed using Fisher’s exact test. Kaplan–Meier curves truncated at 180 days were constructed for the four-group analysis and compared using the log-rank test. An exploratory adjusted Cox model using the four-level exposure variable was also generated as a secondary visual analysis. Because no deaths occurred in the HMB-ONS ≥ 3 months group, this model was considered exploratory only, given the risk of complete or quasi-complete separation and the resulting instability of formal effect estimates.

A two-sided *p*-value < 0.05 was considered statistically significant. Statistical analyses were performed using IBM SPSS Statistics 26.0 (IBM Corp., Armonk, NY, USA).

### 2.6. Ethics Statement

The study was conducted in accordance with the Declaration of Helsinki and was approved by the Research Ethics Committee of Málaga. Written informed consent was obtained from all participants or their legal representatives according to the original study protocol.

## 3. Results

### 3.1. Study Cohort and Baseline Characteristics

**Figure 1:**
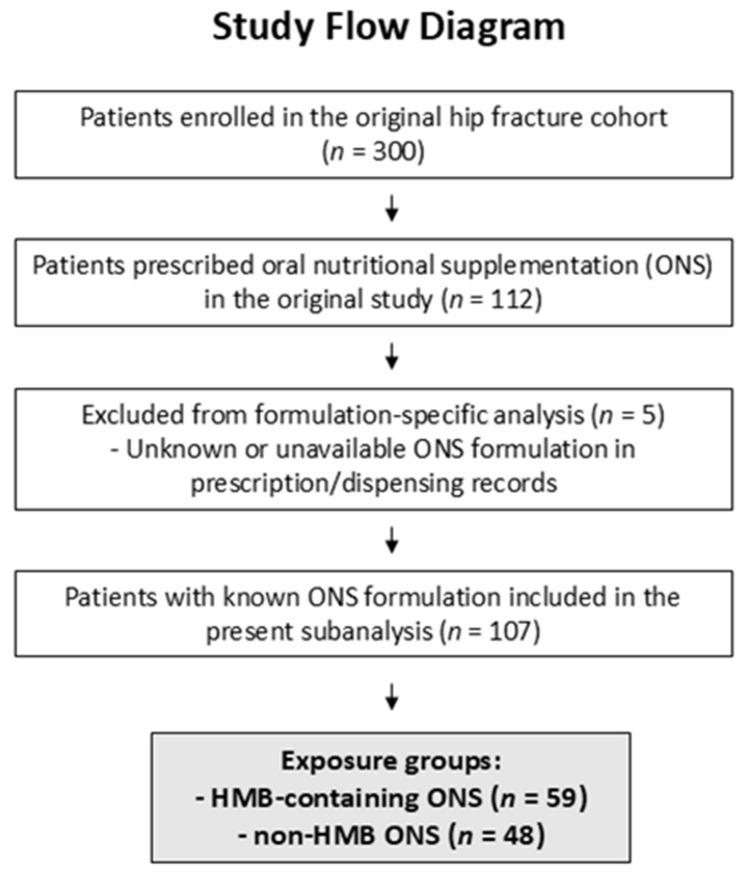
Flow diagram of patient selection for the formulation-specific oral nutritional supplement (ONS) subanalysis.

A total of 107 patients with hip fractures and a confirmed prescription of a known oral nutritional supplement (ONS) formulation were included in this formulation-specific analysis. Patients with unknown or unavailable ONS formulation in the prescription/dispensing records were excluded from the formulation-specific comparison. Among the included patients, 59 received HMB-containing ONS, and 48 received non-HMB ONS (Figure 1). The HMB-containing group consisted of patients prescribed a single high-calorie, high-protein HMB-enriched formulation, whereas the non-HMB group comprised patients prescribed a heterogeneous set of formulations, predominantly diabetes-specific and renal-oriented supplements according to clinical indication. Detailed information on the distribution of ONS formulations is provided in Supplementary Table S1.

The analyzed cohort was composed predominantly of older adults with substantial comorbidity and nutritional impairment after hip fracture, consistent with the characteristics of the broader malnourished post-fracture population from which this subgroup was derived. Both treatment groups shared a broadly similar demographic and nutritional profile, although clinically relevant differences in comorbidity burden and renal function were observed, as detailed in Table 1.

Patients receiving HMB-containing ONS and those receiving non-HMB ONS were of similar age (84.1 ± 6.2 vs. 84.5 ± 6.7 years; *p* = 0.718) and showed no significant differences in length of hospital stay (9.0 ± 7.9 vs. 8.5 ± 3.9 days; *p* = 0.646), body mass index (23.0 ± 4.7 vs. 22.4 ± 4.5 kg/m^2^; *p* = 0.489), serum albumin at admission (2.50 ± 0.45 vs. 2.45 ± 0.39 g/dL; *p* = 0.542), baseline Barthel Index (74.0 ± 27.9 vs. 70.2 ± 29.8; *p* = 0.501), or baseline MNA-SF score (6.39 ± 2.25 vs. 6.27 ± 2.51; *p* = 0.797).

However, patients prescribed non-HMB ONS had a significantly greater comorbidity burden, with a higher Charlson comorbidity index (6.98 ± 1.96 vs. 5.42 ± 1.58; *p* < 0.001), and worse renal function at admission, with lower estimated glomerular filtration rate (46.5 ± 24.7 vs. 67.5 ± 21.0 mL/min/1.73 m^2^; *p* < 0.001). Likewise, diabetes mellitus (68.8% vs. 5.1%; *p* < 0.001) and chronic kidney disease stage ≥ 3b (41.7% vs. 5.1%; *p* < 0.001) were substantially more frequent in the non-HMB ONS group. These findings indicate a clinically relevant baseline imbalance, with the group receiving non-HMB ONS presenting a higher burden of comorbidity and renal impairment.

Overall mortality in the analyzed cohort was 14.0% (15/107) at 3 months, 23.4% (25/107) at 6 months, and 29.9% (32/107) at 12 months.

### 3.2. Main Association Between HMB-Containing ONS and Mortality

Across all evaluated follow-up horizons, mortality was numerically lower among patients receiving HMB-containing ONS than among those receiving non-HMB ONS, with the clearest separation observed at 6 months. In multivariable logistic regression models adjusted for sex, age, and Charlson comorbidity index, the direction of the association remained consistent across all follow-up horizons (Table 2). At 3 months, the association did not reach statistical significance (OR 0.454; 95% CI 0.125–1.644; *p* = 0.229). At 6 months, HMB-containing ONS was associated with lower mortality after adjustment for sex, age, and Charlson comorbidity index (OR 0.267; 95% CI 0.091–0.784; *p* = 0.016). At 12 months, the effect estimate remained directionally favorable but was attenuated and no longer statistically significant (OR 0.502; 95% CI 0.194–1.301; *p* = 0.156).

Based on these findings, 6-month mortality was selected as the focus of the subsequent exploratory time-to-event analyses.

Sensitivity analyses for 6-month mortality are shown in Supplementary Table S2. The association between HMB-containing ONS and lower 6-month mortality remained significant when the Charlson comorbidity index was replaced by diabetes mellitus. When the Charlson comorbidity index was replaced by renal impairment, the association was attenuated and did not reach statistical significance, although the direction of effect was preserved. Furthermore, a sensitivity model including both diabetes mellitus and chronic kidney disease stage ≥ 3b simultaneously yielded results consistent with the primary analysis; HMB-containing ONS remained independently associated with lower 6-month mortality (OR 0.145; 95% CI 0.031–0.672; *p* = 0.014).

### 3.3. Time-to-Event Analysis at 6 Months

Because the strongest association was observed at 6 months, a time-to-event analysis was performed over this interval. In a Cox proportional hazards model adjusted for sex, age, and Charlson comorbidity index, prescription of HMB-containing ONS was associated with a significantly lower hazard of death within 6 months compared with non-HMB ONS (HR 0.358; 95% CI 0.145–0.885; *p* = 0.026). Visual inspection of log-minus-log survival plots did not suggest major violations of the proportional hazards assumption. Similar results were observed in a sensitivity Cox model incorporating diabetes mellitus and chronic kidney disease stage ≥ 3b instead of the Charlson comorbidity index, in which HMB-containing ONS remained associated with a lower hazard of 6-month mortality (HR 0.283; 95% CI 0.084–0.947; *p* = 0.041).

**Figure 2:**
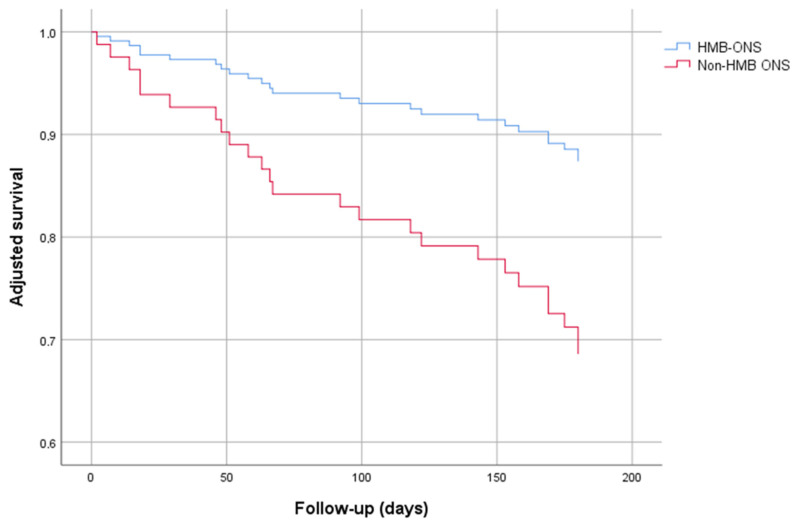
Model-derived adjusted survival curves from the Cox proportional hazards model for 6-month mortality according to oral nutritional supplement (ONS) formulation. Curves were adjusted for age, sex, and Charlson comorbidity index and do not represent raw Kaplan–Meier survival estimates.

Adjusted survival curves showed progressive separation between groups during follow-up (Figure 2), supporting an early survival association favoring HMB-containing ONS during the post-fracture period.

### 3.4. Exploratory Analysis According to Supplement Formulation and Treatment Persistence

At 6 months, mortality differed significantly according to supplement formulation and treatment persistence (Table 3). The lowest mortality was observed in patients receiving HMB-containing ONS for ≥3 months (0/18, 0.0%), whereas the highest mortality occurred in those receiving non-HMB ONS for <3 months (14/34, 41.2%). Patients receiving HMB-containing ONS for <3 months and non-HMB ONS for ≥3 months showed intermediate mortality rates (8/41, 19.5% and 3/14, 21.4%, respectively), resulting in a significant overall difference across the four groups (chi-square *p* = 0.007).

Exploratory pairwise comparisons were consistent with the observed mortality gradient. Among patients treated for <3 months, mortality was significantly lower in the HMB-ONS group than in the non-HMB ONS group (8/41 [19.5%] vs. 14/34 [41.2%], *p* = 0.046). In the comparison restricted to sustained users (≥3 months), mortality was 0.0% (0/18) in the HMB-ONS group and 21.4% (3/14) in the non-HMB ONS group, although this difference did not reach conventional statistical significance (*p* = 0.073). Given the limited sample size and the absence of deaths in the HMB-ONS ≥ 3 months group, these pairwise comparisons should be interpreted cautiously.

### 3.5. Exploratory Survival Analyses According to Supplement Formulation and Treatment Persistence

**Figure 3:**
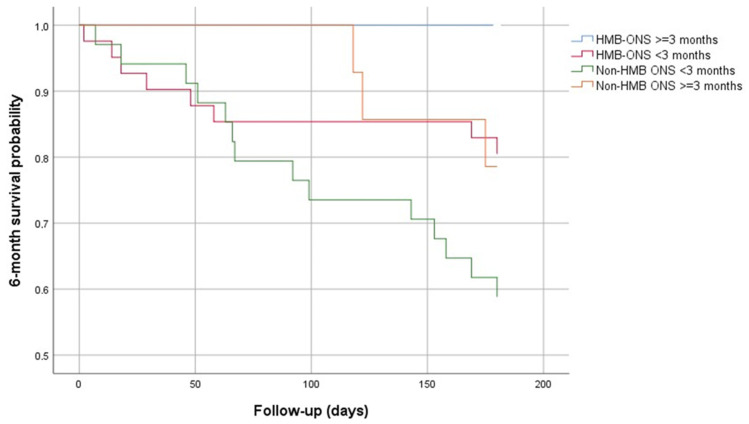
Kaplan–Meier survival curves truncated at 180 days for 6-month mortality according to oral nutritional supplement formulation and duration of use.

Kaplan–Meier survival curves truncated at 180 days showed significant differences in 6-month survival according to supplement formulation and treatment persistence (log-rank *p* = 0.004; Figure 3). The most favorable survival profile was observed among patients receiving HMB-containing ONS for ≥3 months, whereas the least favorable profile was seen in those receiving non-HMB ONS for <3 months. The remaining two groups (HMB-containing ONS for <3 months and non-HMB ONS for ≥3 months) showed intermediate survival patterns.

**Figure 4:**
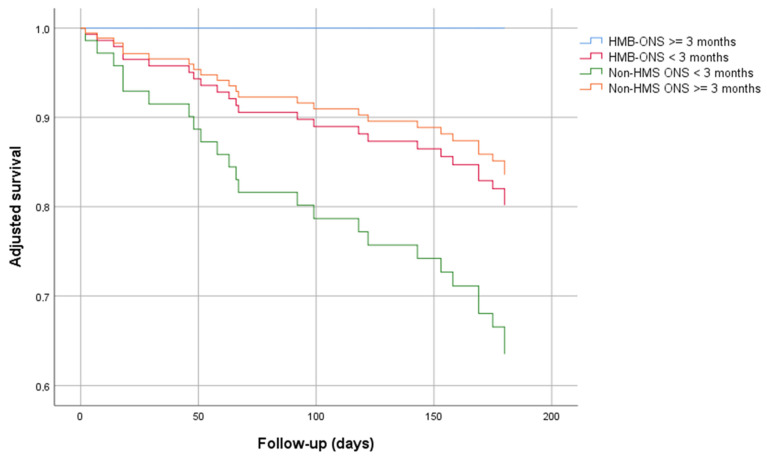
Model-derived adjusted survival curves from an exploratory Cox proportional hazards model according to oral nutritional supplement (ONS) formulation and treatment persistence. Curves were adjusted for age, sex, and Charlson comorbidity index and are presented for visual exploratory purposes only. They do not represent raw Kaplan–Meier survival estimates.

Because no deaths occurred in the HMB-ONS ≥ 3 months group, adjusted regression models using the four-level exposure variable were affected by complete or quasi-complete separation, limiting the stability and interpretability of formal effect estimates. Accordingly, the corresponding adjusted Cox model was considered exploratory and is presented primarily as a visual complement to the unadjusted Kaplan–Meier curves (Figure 4), rather than as a basis for formal inference. Despite this limitation, the exploratory adjusted survival curves remained directionally consistent with the unadjusted Kaplan–Meier analysis, showing the most favorable survival profile in patients receiving HMB-containing ONS for ≥3 months and the least favorable profile in those receiving non-HMB ONS for <3 months.

## 4. Discussion

In this formulation-specific subanalysis of a prospective cohort of malnourished older adults with hip fracture, prescription of HMB-containing ONS was associated with lower 6-month mortality compared with non-HMB ONS. This association was observed in crude analyses, remained significant after adjustment for sex, age, and Charlson comorbidity index, and was supported by the adjusted Cox time-to-event analysis. The direction of the association was consistent at 3, 6, and 12 months, although the effect was most evident at 6 months and attenuated at 12 months. Exploratory analyses combining supplement formulation and treatment persistence showed a marked 6-month mortality gradient, with the lowest mortality in patients receiving HMB-ONS for ≥3 months and the highest mortality in those receiving non-HMB ONS for <3 months. Overall, these findings suggest that, in this real-world cohort, ONS formulation may be clinically relevant in addition to treatment persistence, although the observational design and baseline imbalance between groups require cautious interpretation.

The present results should be interpreted within the broader context of nutritional care after hip fracture. Malnutrition is highly prevalent in this population and has been consistently associated with complications, impaired functional recovery, and mortality [1,2,3,4]. In previous analyses of this same cohort, nutritional status assessed by SGA and MNA-SF showed prognostic value for mortality after hip fracture [3], and phase angle was associated with mortality independently of age and comorbidity [23]. These findings support the concept that nutritional impairment in older adults with hip fracture is not merely a descriptive feature, but a clinically meaningful marker of vulnerability and prognosis. Recent studies have further shown that malnutrition in hospitalized older adults is associated with adverse biochemical, hematological, immunological, oxidative stress, and adipokine profiles, reinforcing its role as a multidimensional marker of biological vulnerability and adverse outcomes [24,25]. In this setting, ONS is a rational intervention [6], but available evidence regarding its impact on survival remains heterogeneous. Randomized and interventional studies have reported improvements in nutritional intake, nutritional parameters, complications, or functional outcomes [9,10,11,12], whereas meta-analyses and reviews have suggested benefits on postoperative complications but less consistent effects on mortality [5,7,8,13]. Our findings add to the literature by addressing a more specific question: not only whether ONS is prescribed, but whether the formulation prescribed may be associated with different survival patterns in malnourished patients.

The stronger association observed at 6 months is clinically plausible. The early months after hip fracture represent a period of high metabolic stress, inflammation, immobility, reduced oral intake, and accelerated muscle loss [26]. During this window, interventions targeting protein–energy intake and muscle preservation may have greater potential to influence recovery, particularly given the relevance of adequate protein intake for muscle function in older adults and the increased protein requirements described in acute or chronic disease [27,28]. By contrast, 12-month mortality is likely to be increasingly influenced by frailty, long-term disability, comorbidity progression, institutionalization, and intercurrent events. Therefore, attenuation of the association at 12 months does not necessarily contradict the 6-month signal, but it does indicate that the effect should not be interpreted as a sustained or definitive survival benefit. Rather, it may reflect an early post-fracture association that becomes diluted over time as competing determinants of mortality accumulate, consistent with previous evidence showing that nutritional interventions after hip fracture may improve selected nutritional, functional, or complication-related outcomes while having less certain effects on longer-term hard endpoints [5,7,8,13,15]. Accordingly, the 6-month findings should be interpreted as hypothesis-generating and require confirmation in prospective studies with pre-specified primary endpoints.

The potential relevance of HMB-containing ONS is also biologically plausible. HMB, a leucine metabolite, has been associated with anti-catabolic and muscle-preserving effects in older or clinically vulnerable populations, which may be particularly relevant in patients exposed to acute immobilization and inflammation after fracture [17,18,19,20,21,29]. In patients with hip fractures, HMB-enriched nutritional supplementation has been associated with improvements in sarcopenia-related parameters, muscle recovery, functional outcomes, postoperative immobilization, or nutritional recovery [17,18,22]. The FracNut study provides recent real-world evidence in an older hip fracture population with malnutrition or nutritional risk receiving a high-calorie, high-protein ONS enriched with HMB, showing favorable effects on nutritional and functional outcomes, although mortality was not the primary endpoint [22]. Our analysis complements these findings by exploring survival in a formulation-specific comparison. However, because ONS formulation was selected according to routine clinical practice rather than randomized allocation, our results should be regarded as hypothesis-generating rather than confirmatory evidence of a causal effect of HMB.

The exploratory analysis according to formulation and treatment persistence is consistent with our previous adherence study, in which sustained ONS use for at least 3 months was associated with lower mortality among malnourished patients after hip fracture [16]. The present subanalysis extends that observation by suggesting that survival patterns may depend not only on treatment persistence but also on the formulation prescribed. The absence of deaths among patients receiving HMB-ONS for ≥3 months is noteworthy and directionally consistent with a potential benefit of sustained exposure to an HMB-containing, high-calorie, high-protein formulation. However, this finding also creates an important statistical limitation, because the absence of events in one subgroup leads to complete or quasi-complete separation and unstable formal estimates. For this reason, the four-group Cox model was deliberately presented as an exploratory visual complement rather than as formal inferential evidence. Moreover, sustained treatment itself may be influenced by survival, tolerance, functional recovery, caregiver support, and healthcare system factors. Patients must also survive long enough to be classified as persistent users, introducing the possibility of survival-related bias (immortal time bias) and reverse causation. Therefore, the 4-group findings should be interpreted as a clinically suggestive pattern, not as proof that maintaining HMB-ONS for ≥3 months causes a reduction in mortality.

A central issue in the interpretation of this study is the imbalance in diabetes and renal disease between groups. Patients receiving non-HMB ONS had a higher Charlson comorbidity index, a much higher prevalence of diabetes mellitus, more frequent chronic kidney disease stage ≥ 3b, and worse baseline renal function. This imbalance is not unexpected in a real-world cohort, because non-HMB ONS included a heterogeneous group of standard and disease-specific formulations, including diabetes- and renal-oriented products. Thus, prescription of non-HMB ONS may partly reflect the clinical profile of patients with diabetes, renal impairment, or other comorbidities. This introduces a clear risk of confounding by indication or channeling bias, in which the same clinical characteristics that influence treatment selection may also influence outcomes [30,31]. This issue is relevant because diabetes has been associated with excess mortality and complications after hip fracture in both cohort studies and meta-analytic evidence [32,33], while chronic kidney disease is highly prevalent among older hip fracture patients and has been associated with higher mortality and worse clinical outcomes [34,35]. In our sensitivity analyses, the association between HMB-containing ONS and lower 6-month mortality remained significant when the Charlson comorbidity index was replaced by diabetes mellitus, suggesting that the observed association is unlikely to be explained solely by diabetes imbalance. Conversely, when Charlson was replaced by renal impairment, the association was attenuated and did not reach statistical significance, although the direction of effect was preserved. However, further sensitivity analyses incorporating both diabetes mellitus and chronic kidney disease stage ≥ 3b simultaneously yielded results consistent with the primary analysis in both logistic and Cox regression models. Taken together, these findings suggest that renal disease and the clinical decision-making linked to renal-oriented supplementation may partly contribute to the observed association, but are unlikely to fully explain it. Overall, the results are compatible with a potential formulation-specific association with survival, although residual confounding related to renal impairment, comorbidity severity, treatment selection, and other unmeasured clinical factors cannot be excluded.

Several methodological aspects strengthen the present analysis. The study was derived from a prospective, well-characterized cohort of older adults with fragility hip fractures, with systematic nutritional assessment and follow-up at 3, 6, and 12 months. The analysis was restricted to patients with known ONS formulation, allowing a formulation-specific comparison that is rarely addressed in this population. The main models were adjusted for age, sex, and Charlson comorbidity index, and sensitivity analyses specifically addressed diabetes and renal impairment, the most clinically relevant sources of potential confounding by indication in this cohort. In addition, treatment persistence was assessed using electronic pharmacy dispensing records, providing an objective real-world measure of ONS retrieval after discharge. Although dispensing does not guarantee actual intake, it is less susceptible to recall bias than self-reported adherence and reflects treatment persistence under routine clinical conditions.

The study also has important limitations. Its observational design precludes causal inference, and ONS formulation was selected according to routine clinical practice rather than randomized allocation. The non-HMB group included heterogeneous formulations, including disease-specific products prescribed for patients with diabetes or renal impairment, and residual confounding by indication remains likely despite adjustment and sensitivity analyses. The sample size was limited, particularly in the exploratory 4-group analysis, and the absence of deaths in the HMB-ONS ≥ 3 months subgroup prevented stable estimation of formal adjusted effects for this exposure category. The analysis was restricted to patients with known ONS formulation, which improves exposure classification but may limit generalizability. ONS persistence was inferred from pharmacy dispensing records, which document supplement retrieval but not actual consumption. In addition, these records did not provide reliable information regarding the daily dose effectively consumed, adherence to the prescribed regimen, or reasons for treatment discontinuation. Therefore, dispensing persistence should be interpreted as a proxy of treatment exposure rather than a direct measure of supplement intake. Finally, the study did not evaluate cause-specific mortality, changes in muscle mass, physical performance, inflammatory markers over time, or functional recovery as mediators of the observed association, and the 6-month focus should be interpreted as the most informative time horizon in this subanalysis rather than as a pre-specified primary endpoint established before examining the data. In addition, several analyses were exploratory, and no formal adjustment for multiple comparisons was performed; therefore, the findings should be interpreted as hypothesis-generating and require confirmation in larger prospective studies.

From a clinical perspective, these findings suggest that ONS formulation may deserve greater attention in the nutritional management of malnourished older adults after hip fracture. The results do not justify concluding that HMB-containing ONS is superior to other ONS formulations in a causal sense, nor do they imply that disease-specific formulations are inappropriate in patients with diabetes or renal impairment. Rather, they indicate that formulation choice, comorbidity profile, and treatment persistence are closely intertwined in real-world nutritional care. Larger prospective studies, ideally with randomized or pragmatic comparative designs, are needed to determine whether HMB-containing high-calorie, high-protein ONS confers survival or functional advantages in malnourished hip fracture patients, and whether such effects differ according to diabetes, renal function, baseline sarcopenia, inflammatory burden, or treatment adherence.

## 5. Conclusions

In conclusion, this formulation-specific subanalysis suggests an association between HMB-containing ONS and lower 6-month mortality compared with non-HMB ONS in malnourished older adults after hip fracture. The association was strongest at 6 months, attenuated at 12 months, and was accompanied by an exploratory mortality gradient according to both supplement formulation and treatment persistence. However, the findings must be interpreted with caution because of the observational design, small sample size, heterogeneous comparator group, and potential residual confounding related particularly to renal impairment and formulation selection. These results support the hypothesis that ONS formulation may matter in the prognosis of malnourished older adults after hip fracture and should be confirmed in larger prospective studies.

## Figures and Tables

**Table 1:** Baseline characteristics of the study cohort.

	HMB-Containing ONS (*n* = 59)	Non-HMB ONS (*n* = 48)	*p*-Value
Age, years	84.07 ± 6.22	84.52 ± 6.68	0.718
Female sex, n (%)	55 (93.2)	39 (81.3)	0.059
Charlson comorbidity index	5.42 ± 1.58	6.98 ± 1.96	<0.001
Diabetes mellitus, n (%)	3 (5.1)	33 (68.8)	<0.001
Chronic kidney disease stage ≥ 3b, n (%)	3 (5.1)	20 (41.7)	<0.001
Previous fracture, n (%)	9 (15.3)	6 (12.5)	0.683
Length of stay, days	9.03 ± 7.89	8.46 ± 3.92	0.646
Body mass index, kg/m^2^	23.02 ± 4.71	22.39 ± 4.55	0.489
Serum albumin at admission, g/dL	2.50 ± 0.45	2.45 ± 0.39	0.542
eGFR at admission, mL/min/1.73 m^2^	67.54 ± 21.03	46.47 ± 24.74	<0.001
Barthel Index at baseline	73.98 ± 27.94	70.21 ± 29.80	0.501
MNA-SF at baseline	6.39 ± 2.25	6.27 ± 2.51	0.797

**Table 2:** Association between HMB-containing oral nutritional supplements and mortality at 3, 6, and 12 months.

Follow-Up Time	HMB-ONS, Deaths/Total (%)	Non-HMB ONS, Deaths/Total (%)	Crude *p*-Value *	Adjusted OR (95% CI) †	Adjusted *p*-Value
3 months	6/59 (10.2)	9/48 (18.8)	0.266	0.454 (0.125–1.644)	0.229
6 months	8/59 (13.6)	17/48 (35.4)	0.011	0.267 (0.091–0.784)	0.016
12 months	13/59 (22.0)	19/48 (39.6)	0.058	0.502 (0.194–1.301)	0.156

* Crude *p*-values were calculated using Fisher’s exact test. † Adjusted odds ratios were obtained from multivariable logistic regression models including sex, age, and Charlson comorbidity index.

**Table 3:** Six-month mortality according to oral nutritional supplement formulation and treatment persistence.

Exposure Group	Deaths/Total	Mortality, %
HMB-ONS ≥ 3 months	0/18	0.0
HMB-ONS < 3 months	8/41	19.5
non-HMB ONS < 3 months	14/34	41.2
non-HMB ONS ≥ 3 months	3/14	21.4

## Data Availability

The datasets presented in this article are not readily available due to technical limitations.

## Supplementary Materials

The following supporting information can be downloaded at: https://www.mdpi.com/article/10.3390/nu18121891/s1, Table S1: Distribution of oral nutritional supplement formulations according to study group. Table S2: Sensitivity analyses for the association between HMB-containing oral nutritional supplementation and 6-month mortality.
