# Supplementary material for: HMB-Containing Oral Nutritional Supplementation and Mortality After Hip Fracture in Malnourished Older Adults: A Formulation-Specific Subanalysis of a Prospective Cohort
Source: Nutrients. 2026 Jun 11;18(12):1891. doi: 10.3390/nu18121891 (PMC13305463; doi:10.3390/nu18121891)
Supplement: Supplementary file 1 [file nutrients-18-01891-s001.zip › nutrients-4352315-supplementary.pdf]

Supplementary Materials

Supplementary Table S1. Distribution of oral nutritional supplement formulations according to study group

ONS formulation category	Specific formulation	n (%)
HMB-containing ONS	High-calorie, high-protein HMB-enriched formulation	59 (100.0)
Non-HMB ONS	Diabetes-specific formulations	26 (54.2)
Non-HMB ONS	Renal-oriented formulations	17 (35.4)
Non-HMB ONS	Other standard formulations	5 (10.4)

Note: ONS, oral nutritional supplement.

Supplementary Table S2. Sensitivity analyses for the association between HMB-containing oral nutritional supplementation and 6-month mortality.

Model Adjustment variables	Adjusted OR	(95% CI)	p value
Main model Sex, age, Charlson comorbidity index	0.267	(0.091–0.784)	0.016
Sensitivity model 1 Sex, age, diabetes mellitus	0.120	(0.032–0.453)	0.002
Sensitivity model 2 Sex, age, renal impairment	0.356	(0.119–1.066)	0.065
Sensitivity model 3 **Sex, age, diabetes,** renal impairment	0.145	(0.031–0.672)	0.014

Note: Odds ratios are shown for HMB-containing ONS compared with non-HMB ONS. Sensitivity models replaced the Charlson comorbidity index with diabetes mellitus or renal impairment as clinically relevant covariates. ONS, oral nutritional supplement; OR, odds ratio; CI, confidence interval.
